# Gender Differences in Healthy Lifestyle, Body Consciousness, and the Use of Social Networks among Medical Students

**DOI:** 10.3390/medicina57070648

**Published:** 2021-06-24

**Authors:** Lavinia-Maria Pop, Magdalena Iorga, Lucian-Roman Șipoș, Raluca Iurcov

**Affiliations:** 1 Faculty of Psychology and Education Sciences, “Alexandru Ioan Cuza” University, 700111 Iasi, Romania; lavinia.pop@student.uaic.ro; 2 Behavioral Sciences Department, “Grigore T. Popa” University of Medicine and Pharmacy, 700115 Iasi, Romania; 3 Dentistry Department, Faculty of Medicine, University of Oradea, 410073 Oradea, Romania; lsipos@uoradea.ro (L.-R.Ș.); riurcov@uoradea.ro (R.I.)

**Keywords:** students, medicine, healthcare, lifestyle, body image, social network, self-esteem, body mass index, physical health

## Abstract

*Background and Objectives*: The goal of this survey was to identify the relationship between the level of satisfaction with body image, perceived health, and the usage of social media among freshmen medical university students. The influence of social media and peers was also related to body image. *Materials and Methods*: An online survey was distributed among freshmen healthcare students. The questionnaire collected sociodemographic, anthropometric data, and information about students’ perception about healthy lifestyle using open-ended questions, as well as their opinion about the importance of perfect body image and the level of satisfaction with their physical appearance. Questions focusing on the use of social media and the relationship with body image collected data on the use of social networks and how they affect students’ opinion about their own body image. Psychometric data were also gathered using the Body Consciousness Scale. For the statistical analysis, QSR NUD*IST (Non-numerical Unstructured Data Indexing Searching and Theorizing) Vivo 12 was used for qualitative data and IBM Statistical Package for Social Sciences (SPSS) Statistics for Windows, version 23 (SPSS Inc., Chicago, IL, USA) was used for descriptive and comparative results. *Results*: In total, 77 students aged 20.09 ± 2.47 years, of which the majority were women (75.30%), were included in the survey. The use of social network was about 4.81 ± 3.60 h/day. Facebook was the most used social networking site (94.80%), followed by Instagram (92.20%), Snapchat (16.90%), WhatsApp (15.60%), and TikTok (10.40%). The most common reason for using these sites was socialization. We found that 64.90% of healthcare students were normal weight. The main barriers for having a healthy lifestyle, as they were perceived by students, were the busy schedule and the lack of time needed to prepare healthy meals, lack of motivation, and lack of money. Women scored higher for the Private Body Consciousness and Public Body Consciousness scales. The main aspects related to a healthy lifestyle referred to physical activity, consumption of fruit and vegetables, water consumption, and a good quality of sleep. Gender differences were discussed as well. *Conclusions*: The results illustrated the complexity of the relationship between social media and body image and the need to prevent body image concerns, especially in young women.

## 1. Introduction

Lifestyle is defined as a pattern of behavior, the attempt to ensure optimal health, or the sum of health-related habits that have a positive effect on overweight and obesity [1,2]. In the current study, lifestyle was considered by the researchers as a key factor in health status, owing to the statement by the World Health Organization (WHO) that 60% of factors related to individual health and quality of life are correlated with lifestyle [3].

According to current data, lifestyle is based on personal choices and identities and has a significant influence on the physical and mental health of the people [3]. At the micro level, the personality, biological, and psychological characteristics of the individual, family, friends, school, and society affect the daily life and lifestyle of the individual. At the macro level, the city and the environment in which the individual lives, the media, and the cultural climate of the individual‘s society all affect the lifestyle and changes in the life of the individual [4].

According to the WHO, late adolescence (from 19 to 24 years) is an important stage in a person’s development and is defined as a period in which the individual prepares for work and assumes adult responsibilities [5]. The basis of a healthy lifestyle is built during youth, when eating habits develop intensively and body weight and body image become correlated [6]. Lifestyle, weight, body image, and satisfaction define the physical and mental health status during adulthood. An unhealthy diet during the period of intense growth can affect the development of young people, triggering the body’s nonacceptance and causing diet-related diseases in adulthood [7].

During the period of their academic studies (late youth), students should be physically and mentally healthy, practicing an active life and having healthy eating behaviors to maintain their health status. They need a good physical and mental condition to cope with the effort required during their academic years and to have a satisfactory body image [5,8]. A poor perception of well-being can lead to less involvement in good self-care practices or poor adherence to interventional programs, while a perceived good or excellent level of well-being is associated with good personal care [9].

Body image has several components, which can be divided into two dimensions: (a) Perceptual (how we see our size, shape, weight, face, movement, and actions) and (b) attitudinal (how we feel about these traits and how these feelings affect our behaviors) [10]. Body image includes the thoughts, beliefs, and evaluation of emotions and behaviors about the physical appearance of an individual, all of which are affected by multiple factors. To improve their mental body image, people resort to different methods, including various diets that promise weight loss, exercise, or even cosmetic surgery [11].

Researchers have identified that healthcare students have an increased focus on their own health, practice a better lifestyle compared to other student populations, and have a favorable perception of their own health and body [12,13]. The strong relationship between healthy lifestyle, body image, and healthy behaviors is interrelated with mental health [9]. Body image and beauty are some of the main causes of stress among young people [10]. However, on the other hand, healthcare students have also been shown to experience high levels of stress related to their academic tasks, which is a causative factor in eating disorders [14]. The sedentary lifestyle and stressful nature of medical student activity seems to prevent them from maintaining a positive perception of body image [15].

Body dissatisfaction and exposure to the thin-ideal image are major predictors of the development of an eating disorder, the latter having significant health implications. Students experience psychological and social changes, which are more pronounced in subjects experiencing considerable social pressures [11]. Thus, the standards of cultural beauty, as communicated by the media, become messages with a strong impact both for women and men, leading to the development of unhealthy weight control practices [16].

In the Western European countries, body image has become increasingly important, with images of movie stars and fashion models having a strong impact on girls’ body shape and image perception. Such means of mass communication and various sociocultural pressures are considered to cause an increased awareness of being thin as an ideal and contribute to the misperception of body weight [17]. Media has a significant impact on body image as a powerful force that uses various aspects, including the internet, television, magazines, video games, and smartphones to present the ideal of beauty [8]. Currently, media promotes a slim figure model among women and muscular/athletic figure among men, promoting foods and diets with inadequate nutritional value, which can also lead to unwanted nutritional and health behaviors and the nonacceptance of body weight [7]. Both women and men often choose to expose themselves to idealized body images as presented in the media. There is a two-way relationship between exposure to a thin-ideal environment and body dissatisfaction: People who are dissatisfied with their appearance address the media that present thin and beautiful models, possibly for advice or information or to see advertising products designed to bring the aspects of the appearance closer to the perceived ideal [16].

The influence of advertising on social networks and its effect on young people’s self-image has been studied [18,19]. Social platforms are often used by students for communication (social life), as well as for academic tasks [20]. Social platforms such as Instagram, Pinterest, and Facebook are extremely popular. Some social media platforms—such as Instagram—are more visual/image-based compared to others—such as Facebook [21]—and have great impact on body consciousness (awareness of observable aspects of body). A person’s awareness of her/his failure to meet these standards leads to dissatisfaction, and media is seen as particularly important promotor of the ideal body image.

In the last decade, social networks have become the most common form of sociocultural interaction for young people due to various factors such as communicating with peers, maintaining contact with friends and family, and sharing information. On social networks, people often present an idealized version of themselves by uploading only the most attractive images of them (which can be edited and enhanced) in their profile and removing any images they find unattractive. Although social networks contain images of several different types of people (e.g., friends, family, strangers, celebrities), these are generally used to interact with colleagues. Finally, in addition to images, people often post other content related to appearance and comments on social networks, which could also impact users’ feelings about their appearance [22].

However, among students, it has been shown that comparison to other colleagues’ pictures and posting on social media influences self-satisfaction with the body image considerably. Body dissatisfaction and exposure to thin-ideal images are major predictors for eating disorders. Social networks have a strong influence on young people, even changing their habits and lifestyles [23,24]. Some studies conducted on students have shown that body worship currently prevails among college students, and it is impossible to deny the influence of many factors such as food habits, social pressure, the aesthetic of thinness, and the popular models on social media and social networks [25,26].

The goal of the present study was to explore health-related beliefs, body image perception, and influence of social networks on the ideal body image among freshmen healthcare students enrolled in kinesiotherapy studies with a focus on gender differences. The study is part of a larger one focusing on health status, body image, and the impact of social media on the ideal body among healthcare students in Romania.

## 2. Materials and Methods

### 2.1. Study Population

The questionnaire was sent to 90 freshmen students enrolled in Balneo-Physio-Kinesiotherapy specialization. The respondents were informed about the purpose of the study and the confidentiality of data. No incentive was given to the participants. Students were informed that they could withdraw from the study whenever they wanted without consequences. The inclusion criteria were students enrolled in their first year of study and surveys returned before deadline. Criteria for excluding surveys from the research were incomplete questionnaires or questionnaires submitted after the deadline. Finally, 77 questionnaires were included in the research.

### 2.2. Data Collection

The questionnaire was created using Google Docs and was distributed online during March–April 2021. The qualitative research design was developed to obtain detailed information about students’ perceptions of the following aspects, namely healthy eating, body image, and the influence of social media.

This study used a triangular research method which included drawing, free association method, and open-ended question surveys. The study collected information regarding the following issues:

− The first part of the questionnaire gathered sociodemographic and anthropometric data (age, gender, marital status, environment, members of the household, average monthly financial income, weight, height, body mass index).
− The second section contained items evaluating students’ perception about healthy lifestyle. The items were constructed as an open-ended question (such as changes considered necessary in students’ eating behavior, reasons why a healthy lifestyle is needed, reasons why they practice/would like to practice physical activity, aspects that prevent them from maintaining a healthy lifestyle). One item referred to the free association method and asked students to write the first 5 words that came to their mind when they thought of a healthy lifestyle.
− The third part of the questionnaire addressed the Body Consciousness Scale developed by Miller, Murphy, and Buss in 1981 [27]. This instrument is a self-report tool that includes 3 subscales related to private body consciousness (awareness of internal sensations), public body consciousness (awareness of observable aspects of body), and body competence. Respondents had to indicate their level of correspondence with the 15 statements using a 5-item Likert-type scale. Answers included “extremely uncharacteristic”, “uncharacteristic”, “neutral”, “characteristic”, and “extremely characteristic”. The minimum score possible for each question is 0 and the maximum is 4. Subjects’ scores were calculated on each separate scale by adding each item’s score and forming 3 subscale composites.
− The fourth section included items related to body image and aimed to identify the main characteristics that students associated with their body, the opinion about the importance of body image today, and the level of satisfaction with their body image. The answers were rated on a Likert-type scale from 1 to 10, where 1 = very dissatisfied and 10 = very satisfied.
− The fifth section comprised questions focusing on the use of social media and the relationship with body image (the use of social networks, the frequency, the reason why these were used, the impact these had on students, the emotional state created by following the posts of colleagues or celebrities, the influence of social networks in students’ tendency to compare themselves with other people, the influence of social networks in the way students perceived their own body, etc.).
− In the sixth section, students were asked to open a separate document, draw the first thing that came into their mind when they thought of a healthy lifestyle, and upload that document in the space provided within the questionnaire.

### 2.3. Statistical Analysis

The data were analyzed using a qualitative data analysis program, QSR NUD*IST (Non-numerical Unstructured Data Indexing Searching and Theorizing) Vivo 12, also called NVivo. To present the sociodemographic characteristics of the samples, we performed analyses using IBM Statistical Package for Social Sciences (SPSS) Statistics for Windows, version 23 (SPSS Inc., Chicago, IL, USA). Results for descriptive statistics were expressed as means and standard deviations (SD), frequencies, and percentages. The same library was used to report the results of the Body Consciousness Scale. A qualitative analysis (word cloud) of the students’ personal perception of a healthy lifestyle was created using the Orange software [28]. Using the same software, a hierarchical clustering analysis was used to compute the similarity of the images sent by the participants regarding their perception of a healthy lifestyle. The Body Mass Index (BMI) calculation was carried out according to WHO guidelines, using standards for the European population: A BMI < 18.5 kg/m^2^ was categorized as underweight, 18.5–24.9 kg/m^2^ as normal weight, 25.0–29.9 kg/m^2^ as pre-obese, 30–34.9 kg/m^2^ as obese class I, 35.0–39.9 kg/m^2^ as obese class II, and ≥40 kg/m^2^ as obese class III [29].

### 2.4. Ethical Approval

The present study was conducted in accordance with the Declaration of Helsinki, and the protocol was approved by the Ethical Committee of Faculty of Medicine, University of Oradea, Romania, with the registration number No. 26214/18.11.2020.

## 3. Results

### 3.1. Sociodemographic and Anthropometric Data

The respondents were students enrolled in the first year and studying kinesiotherapy specialty. Students came from rural and urban areas, aged M = 20.09 ± 2.47 (with a minimum of 18 and a maximum of 39 years old). Most of them were female students (75.30%). Body Mass Index (BMI) was also calculated, conforming to WHO guidelines. The detailed information about sociodemographic and anthropometric data are presented in Table 1.

### 3.2. Overview of the Questionnaire Results

The results of this survey present findings concerning different aspects of students’ life, perceptions about health and body image, and the use of social networks. The themes that emerged from the present research are presented below as headings and supported by quotes from the participants. Each quote is accompanied by the identification number assigned to the participant (participant ID), sex, and age of the participant in parentheses.

#### 3.2.1. Theme 1: Personal Reflections of a Healthy Lifestyle

**Figure 1:**
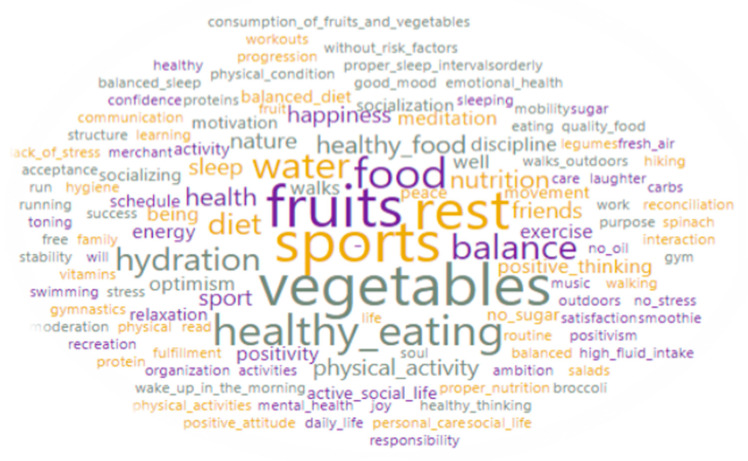
Word cloud of the students’ reflections on the healthy lifestyle.

The first question in the second section of the survey invited students to reflect. They were asked to mention, in a few words, what their perception of a healthy lifestyle was. The results obtained are shown in Figure 1.

As shown in the word cloud visualization, where the size of the word indicates the frequency of its use, the most used words were “sports”, “vegetables”, “fruits”, “healthy eating”, “balance”, “healthy food”, “nutrition”, and “diet”. The importance of hydration, sleep, and rest are highlighted as the main components of a healthy lifestyle through words such as “water”, “hydration”, “sleep”, “rest”, “relaxation”, “proper sleep”, “balanced sleep”, and “high fluid intake”. The students also focused on mental and social health, mentioning various words that refer to these components of a healthy lifestyle, such as “happiness”, “friends”, “optimism”, “positive thinking”, “positive attitude”, “positivity”, “meditation”, “motivation”, “active social life”, “socialization”, “healthy thinking”, “walks”, “outdoors walks”, “nature”, “mental health”, and “soul”.

Regarding the reasons why a healthy lifestyle is necessary, most students scored an increased quality of life, physical, mental, and social well-being and lack of diseases: *A healthy lifestyle is necessary starting with the health of the physical body, to the mental, emotional, spiritual well-being* (participant ID 9: Female, age 20); *Through a healthy lifestyle we stay in shape, we will have a longer and happier life* (participant ID 23: Male, age 20); *The main reason why I consider it is necessary to have a healthy lifestyle is to avoid certain diseases, to maintain a general state of well-being* (participant ID 72: Female, age 20).

Some of the students highlighted the relationship between a healthy lifestyle and physical appearance or body shape: *A healthy lifestyle is necessary because in this way we can maintain both physical and mental balance of the body. Moreover, a healthy lifestyle gives us the opportunity to have a pleasant physical appearance* (participant ID 8: Male, age 19); *For physical appearance, implicit self-esteem and health* (participant ID 71: Female, age 20); *You feel much better in your body, it helps to have a better physical and mental condition* (participant ID 41: Male, age 23); *Because it helps us look better, but in addition our body will feel much better, and this can also help us in emotional states* (participant ID 50: Female, age 21).

At the same time, practicing sports, as another important component of lifestyle, helps to maintain a good physical condition, a harmonious body, to lose weight, and to increase muscle mass, as evidenced by the participants’ answers: *To lose weight, to keep fit, for immunity* (participant ID 10: Female, age 19); *To develop muscle mass* (participant ID 25: Male, age 19); *For better physical condition, optimal body weight, better mood* (participant ID 68: Female, age 19).

#### 3.2.2. Theme 2: Changes in Lifestyle

Most students acknowledged that they had an unhealthy eating behavior, characterized by high consumption of fast food, high carbohydrate intake, low consumption of fruits and vegetables, and other unhealthy behaviors: *I should eat fewer sweets, I should quit smoking, I should practice more sports* (participant ID 70: Female, age 19); *I should reduce sedentary lifestyle, eat a lot more fruits and vegetables, be more organized with the time I eat, and drink less coffee* (participant ID 46: Female, age 19).

Some students mentioned that they would change these things in their lifestyle but face certain obstacles, most often highlighted by the lack of time, busy schedule, lack of motivation, and sometimes money: *Lack of motivation and will, laziness, insufficient time* (participant ID 27: Female, age 20); *The busy schedule and the responsibilities of some days, job and college* (participant ID 60: Female, age 23); *Time, money* (participant ID 73: Female, age 19).

#### 3.2.3. Theme 3: Perception of Body Image

The students were asked to mention three characteristics that they considered most important when thinking about their body and the way it looked. Thus, the most used words were “fit” (*N* = 14), “slender” (*N* = 13), “sensitive” (*N* = 9), “beautiful” (*N* = 9), “small” (*N* = 12), “acceptable” (*N* = 12), “healthy” (*N* = 11), or “cared” (*N* = 6).

The level of satisfaction with body image measured on a Likert-like scale from 1 to 10 (1 = very dissatisfied and 10 = very satisfied) showed that most students were satisfied with the way they looked (M = 7.61 ± 1.57). The Spearman correlation analysis showed that the level of body satisfaction correlated negatively with BMI: The higher the BMI, the lower the satisfaction related to body shape (r = −0.395 **, *p* < 0.001).

Students were also asked to express their opinion about the importance of body image in today’s society and culture. Most believe that beauty standards have been created by culture and imposed by social media: *The body image is important for having success in business, personal and social life* (participant ID 52: Female, age 19); *This is what people see for the first time, plus the media promotes so-called perfection and people tend to frame people in those patterns* (participant ID 65: Female, age 20); *Body image is important only for that person and not for those around them, however it influences relationships between people* (participant ID 37: Female, age 19).

Some other male students considered that the body image is not important: *The body image steals glances, as it were. If you do not have an attractive body image, people will look at you differently, at least that is what I realized and saw in my case, but that is another story. I have been satisfied with myself for many years and people’s opinions no longer matter* (participant ID 28: Male, age 21); *I do not necessarily think that body image is important, for me everyone must feel good in their body no matter what the world says* (participant ID 19: Male, age 20).

#### 3.2.4. Theme 4: Use of Social Media

Although the participants mentioned the use of several social networks, the results show that Facebook was the most used among students (94.8%, *N* = 73), followed by Instagram (92.2%, *N* = 71), Snapchat (16.9%, *N* = 13), WhatsApp (15.6%, *N* = 12), TikTok (10.4%, *N* = 8), YouTube (13%, *N* = 10), Pinterest (5.2%, *N* = 4), and Discord (1.3%, *N* = 1), with students spending an average of M = 4.81 ± 3.60 h/day on these networks. The most common reasons why students used social networks were for socialization (54.6%, *N* = 42), entertainment (26%, *N* = 20), an information source (*N* = 16.9%, *N* = 13), or a way to relax (2.6%, *N* = 2).

For the most part, students stated that social media and peer posts did not influence how they felt about their own body and did not influence the tendency to compare themselves with others. Some of the female students stated that they were affected by these posts in a negative way, leading them to feel insecure and uncomfortable in their own body or becoming envious, while others felt motivated to make a change about the way they looked: *It does not affect me at all. I know my value.* (participant ID 1: Female, age 20); *Sometimes it makes me feel uncomfortable in my body, but most of the time it does not affect me* (participant ID 47: Female, age 19); *Sometimes it motivates me to reach the stage where I am satisfied with my body* (participant ID 68: Female, age 19); *I don’t compare myself to anyone, I’m my only competition* (participant ID 2: Female, age 18); *After I started maturing, they didn’t influence me at all. When I was younger, they had an extremely great influence* (participant ID 61: Female, age 20).

The impact of social networks on body image was found to be seen differently by the respondents. Students considered that social networks had a negative consequence on their psychological status, while some others mentioned that the body-image standard imposed by the social media helped them motivate themselves to become more careful with their weight. Many students agreed that significant time was invested in social networks to the detriment of the individual study program, of the physical activity, or of the effective face-to-face socialization: *It increases the desire to socialize and the need to express yourself freely* (participant ID 8: Male, age 19); *Some people think that it is not a very positive impact, because many people are disrespectful when they see such people, but many of these stars should motivate us to become our best version* (participant ID 22: Female, age 20); *It helped me develop my level of knowledge, I found information that I would not have found* (participant ID 57: Male, age 19); *At least, sedentary lifestyle occurs because we keep looking on social networks and physical activity remains forgotten* (participant ID 28: Male, age 21); *The impact may be different for each person. It may not affect me at all while other people are trying to change because they want to be appreciated and integrate into the standards that are being promoted* (participant ID 37: Female, age 19); *As for me, these social networks kidnap a lot of my life* (participant ID 72: Female, age 20).

### 3.3. Body Consciousness Scale (BCQ)

A three-factor structure of BCQ indicates three dimensions of this scale:

- Private body (representing the disposition to focus on internal bodily sensations)—M = 13.19 ± 2.89,
- Public body (which involves a chronic tendency to focus on and be concerned with the external appearance of the body)—M = 17.68 ± 3.22,
- Body competence (which refers to effective body functioning)—M = 9.83 ± 2.13.

The correlation analysis showed that there was a negative correlation between students’ age and public body subscale (r = −0.380 **, *p* = 0.001): The older the students, the lower their desire to be concerned on with the external appearance of the body.

For the present research, the Cronbach Alpha score was 0.75.

Table 2 presents the results according to gender for the three BCQ subscales.

### 3.4. Drawings

The last item of the survey asked students to open a separate document and draw a picture that came to mind when thinking about a healthy lifestyle. This requirement was doubled by a previous item that asked students to mention, in a few words, what their point of view about a healthy lifestyle was. Thus, analyzing the previous Figure 1, which reflects the themes used by the students to represent a healthy life, we can see that the main aspects related to the positive lifestyle were used (physical activity, fruit and vegetable consumption, water consumption, quality of sleep).

**Figure 2:**
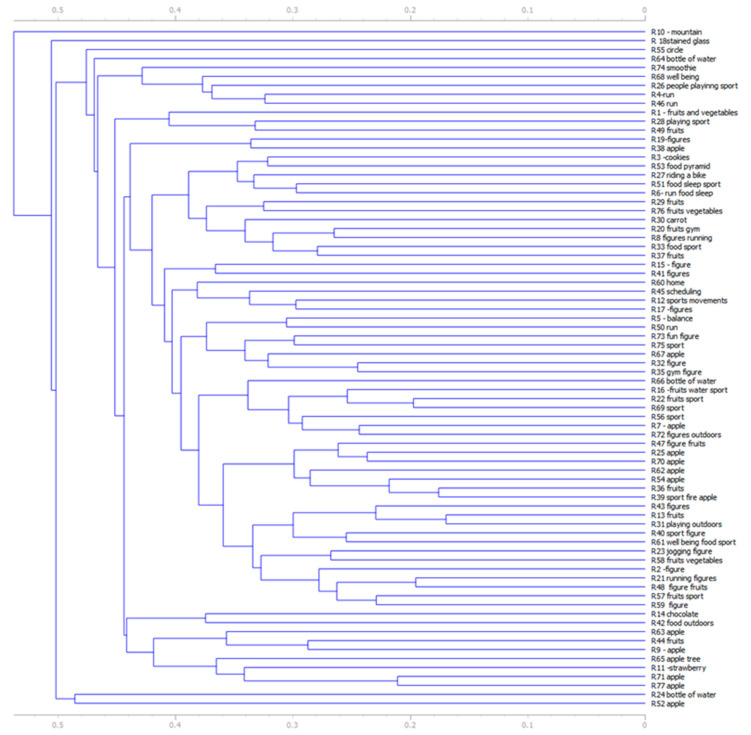
Hierarchical clustering on images that present a healthy lifestyle (R = respondent).

A hierarchical clustering analysis was used to compute the similarity of the images and is presented in Figure 2.

## 4. Discussion

This study presents a qualitative approach with a significant contribution to the inherent aspects of body image, perceived health, and the usage of social media among freshmen medical university students. At the same time, this study provides updated data on the association between self-image and students’ opinions related to the relationship between body-image and social ideals, showing how social media and peers may influence this relationship.

The relationship between body image and healthy lifestyles has been identified in many studies [30,31,32], which have shown that young people dissatisfied with their body might also have poorer health habits. However, few qualitative designs have identified this relationship among medical students.

In our study, we identified a direct relationship between the importance of maintaining a healthy lifestyle and physical appearance. We found that students considered that a healthy lifestyle was necessary because it offered the possibility to have a pleasant physical appearance, to look better, and, implicitly, to have a high self-esteem. A positive association between healthy eating habits and normal body image perception has also been observed in other studies [33,34], which have shown that body image is an important factor for healthy body weight [35].

Students associated a healthy lifestyle with physical activity, eating behavior (which includes eating fruits and vegetables and having a balanced diet), as well as rest and hydration. Although, in theory, they knew the guidelines for following a healthy lifestyle, many of them acknowledged that they would have to improve many aspects to reach a positive lifestyle (namely, reducing the consumption of fast food, carbonated beverages, increasing the consumption of fruits and vegetables, increasing the level of physical activity, etc.). Our results are different from others conducted on students, in general, showing that they failed to achieve a healthy lifestyle due to the practice of diets, lack of physical activity, consumption of cigarettes, and high level of stress [36]. In addition to the results reported by Sogari et al. [37], which showed that students considered “healthy eating” as something related to a lifestyle with positive consequences on the general mentality of the individual, and the concept of “being healthy” as referring to physical and mental health, the questioned students stated that following a healthy lifestyle was necessary for well-being, social health, lack of disease, and overall, an increased quality of life.

The results of the present survey show that the most important barriers for a heathy lifestyle perceived by students were the busy schedule and lack of time needed to prepare healthy meals, lack of motivation, and lack of money. Some studies have reported similar findings, showing that the most common factors perceived as barriers to a healthy diet were time constraints, high food prices, and availability, followed by a lack of motivation in food preparation, which is intricately linked to intention, the main factor in predicting behavior regarding the consumption of healthy foods, such as fruits and vegetables [38,39,40,41]. As the main barriers to a healthy lifestyle that students mentioned, Hilger et al. [42] reported the short time for cooking meals due to academic schedule, some hedonic behaviors (taste of healthy food perceived as unpleasant), the lack of healthy food on campus, and the high cost of healthy food.

BMI values are useful predictors for the risk of bodily dissatisfaction, as some authors have identified [11,43], with younger women being more affected than men [44]. Our results are consistent with these findings, as we identified a negative correlation between BMI and level of body satisfaction among students for both sexes. Most students in this study had a normal BMI (64.9%, *N* = 50), while the rest of the participants were underweight (14.3%, *N* = 11), overweight (15.6%, *N* = 12), or fell into the first class of obesity (5.2%, *N* = 4). However, on a Likert scale from 1 to 10, most participants in our study were quite satisfied with the way they looked. Similar to our results, some other studies conducted on students showed that, in general, most students had a normal weight [45,46], although recent studies on young Europeans have shown increasing trends in overweight and obesity [47,48]. However, contradictory with our results, scientific evidence showed that body image was negatively perceived by most of the women [43]. As comparative results, the study of Aparicio-Martínez [49] identified a higher degree of body satisfaction in men than in women. Aparicio-Martínez also found that body image was focused on obtaining a similar image to that presented by social networks, with analogous findings identified in both men and women subjects.

The scores for the three BCQ subscales proved that women had high scores on the Private Body Consciousness and Public Body Consciousness scales, similar to another study showing that women scored higher on Public Body Consciousness [27] and were more prone to be vulnerable to body image concerns. In our study, a negative correlation was identified between age and public body subscale, which is not consistent with the findings of another study which showed that healthy older people are more aware of external physical appearance and are more positive in self-assessment of body competence than young subjects [50].

The students in our survey mentioned Facebook as the most used social networking site (94.8%, *N* = 73), followed by Instagram (92.2%, *N* = 71), Snapchat (16.9%, *N* = 13), WhatsApp (15.6%, *N* = 12), and TikTok (10.4%, *N* = 8), with the most common reason for using these sites being socialization. There is evidence that social media and internet use are associated with concerns about body image and eating disorders, especially in women, but the causal direction between specific social media and body dissatisfaction cannot be clearly highlighted [51]. A particular type of social media is represented by social networking sites (SNS) such as Facebook, Snapchat, and Instagram that allow users to create public or private profiles and form a network of “friends” or “followers” [52]. Of these networks, Facebook is the most popular social networking site, with over two billion monthly users worldwide in 2017. In addition, in 2016, about 98% of Western students reported having a Facebook account [53]. Instagram (a social networking service exclusively for sharing photos and videos) has grown dramatically in popularity, with over 600 million active users sharing over 95 million photos per day. Instagram is the second most used SNS in Western countries after Facebook [54].

We identified that students spent M = 4.81 ± 3.60 h/day on these networks. Consistent with our finding, another recent study showed that most respondents used social media for 4–6 h a day [55]. Moreover, most medical students (55%) spent 1–4 h a day on social media, and 23% of them spent more than 4 h daily [56]. Moreover, women accessed social networking sites between 6–10 h a day, more than men who spent between 1–5 h on social networking sites every day [57].

The large number of images posted on some social platforms provides regular opportunities for users to make social comparisons related to appearance, with research showing that regular comparison of appearance with others (especially with those seen as more attractive than oneself) can lead to a negative image of one’s own body [58]. Thus, physical aspect comparisons seem to play an important role in the relationship between the use of social networks and concerns about body image [59]. Our results do not fully confirm the findings of these studies related to the influence of social media on the students’ body. Some participants said they knew their own value or were mature enough not to be influenced by posts about the perfect body promoted by social media, while others felt motivated to make the change they wanted in the shape of their body, or on the contrary, they felt uncomfortable in their own body.

However, the participants of this study stated that social networks had both a positive impact on their lives. Social networks increased the desire for free expression and helped students develop knowledge or even motivation by overcoming their own condition regarding physical appearance. However, social networks also had a negative impact by creating addiction, wasting time, or negatively affecting self-image.

Alperstein [60] showed that sharing idealized female images on networks such as Pinterest can contribute to feelings of inadequacy or upward social comparison. Another study among female college students specifically examined the maladaptive use of Facebook (which included seeking negative social assessments from others and generalizing social comparisons), and found that this type of use was associated with increased body dissatisfaction at 4 weeks later [61]. Congruent with these results, Wang et al. [24] identified that individuals presenting a need for social network popularity were more likely to be affected by selfie-viewing behavior in terms of life satisfaction and self-esteem compared with individuals presenting a low need for popularity. According to our results, Heiman and Olenik-Shemesh [62] identified that more women reported higher dissatisfaction with their body appearance, and their parents’ remarks about bodies had an ongoing effect and significant influence on their body self-perceptions.

Social media has the potential to allow more fluidity in gender expression but it also has the potential to perpetuate stereotypes, including beauty standards and body ideals [63]. Social networks that students prefer effectively support their education, for example, YouTube can be an education support channel where teachers find interesting and useful videos or upload ones, and information can be shared on Instagram [64].

The relationship between the promoted image about thinness on social networks and eating disorders and dissatisfaction with body-image has been identified by several studies. For example, Jiotsa et al. [65] revealed that the widespread use of social media in adolescents and young adults was associated with an increase level of dissatisfaction as well as the drive for thinness, therefore rendering subjects more vulnerable to eating disorders. Apacio-Martienz et al. [49] showed that disordered eating attitudes were strongly linked to self-esteem, body image, the desired body, and the use of social media, especially among female subjects. Baceviciene et al. [66] identified the relationship between satisfaction with body image and quality of life among college students. Ansari et al. [67] pointed out that high BMI and depressive symptoms were found more often among students dissatisfied with their weight.

## 5. Strengths and Limitations of the Study

This study brings important results regarding body image, body satisfaction, and the impact on social media on contentment with body image among healthcare students from their first year of study. Another strength is represented by the fact that quantitative and qualitative results are presented, covering a gap of information about students enrolled in kinesiotherapy studies. Third, the results reveal gender differences in perception about body image.

The limitations of the study are represented by the small number of subjects involved in the research and the fact that results cannot be generalized for all categories of students enrolled in different medical specialties. Also, the alpha Cronbach score for the BCQ scale must be evaluated with precociousness, as multiple studies have obtained, in general, a low alpha Cronbach score between 0.59–0.78 [50,68,69].

## 6. Reflections and Planning

The results of the present study highlight the relationship between body image, the influence of social networks, and the importance of a healthy lifestyle on students’ quality of life. Due to the utility of the findings, such information should be disseminated among students, especially to those from medical sciences who are the promoters of a healthy lifestyle and those who will work on providing healthcare to people in need. The impact of these relationships on the satisfaction with body image and the impact on both physical and mental health are important, especially on subjects at this age. In addition, because healthcare students will work with teenagers and young adults, it is useful consider factors such as satisfaction with physical appearance, ideal body image, and the impact of media and social media on health status. The relevant relationship between the perception of one’s physical appearance and depression [70], stress [71], intimate relationship [72], eating disorders [73], food addiction, body mass index [46,74], mental health [75], orthorexia nervosa [76], perfectionism dimensions and physical appearance [77], body image distortions [78], and loneliness [72] are important for both the personal and professional lives of healthcare students.

## 7. Conclusions

The results of the present study illustrate the complexity of the relationship between social media and body image among healthcare students. Furthermore, they emphasize the need to prevent the concerns on the individual’s concept of one’s body, especially in young women. Because of the important impact on physical and psychological health, as well as on practices related to healthy lifestyle, these findings are useful for students, healthcare workers, and university staff to provide better assistance to students and to encourage a high quality of life and satisfaction during academic years.

## Figures and Tables

**Table 1:** Sociodemographic and anthropometric data ^1^.

Sociodemographic and Anthropometric Characteristics	M ± S.D and %
Age	20.09 ± 2.47
Gender	
Male	19 (24.7%)
Female	58 (75.3%)
Environment of origin	
Urban	42 (54.5%)
Rural	35 (45.5%)
Marital status	
Married	4 (5.2%)
Unmarried	73 (94.8%)
Members of the household	
Single	12 (15.6%)
Colleagues/friends	41 (53.2%)
Partner	9 (11.7%)
Parents	15 (19.5%)
Average monthly financial income	
20–100 €	28 (36.4%)
100–200 €	26 (33.8%)
200–400 €	15 (19.5%)
>400 €	8 (10.4%)
Weight	64.58 ± 14.75
Body mass index	22.14 ± 3.50
Nutritional status	
Underweight	11 (14.3%)
Normal weight	50 (64.9%)
Overweight	12 (15.6%)
Obese class I	4 (5.2%)

^1^ Means and standard deviations (M ± S.D), frequency, and percentages (%).

**Table 2:** Gender differences for BCQ Scale ^1^.

Subscales	Men	Women	Differences
Private Body Consciousness	11.00 ± 3.16	13.91 ± 2.42	*p* = 0.000
Public Body Consciousness	15.31 ± 3.36	18.46 ± 2.79	*p* = 0.000
Body Competence	9.26 ± 2.49	10.01 + 1.99	*p* = 0.183

^1^ Means and standard deviations (M ± S.D), frequency, and percentages (%).

## Data Availability

The data presented in this study are available on request from the corresponding author.
